# Rheumatic heart disease awareness in the South West region of Cameroon: A hospital based survey in a Sub-Saharan African setting

**DOI:** 10.1371/journal.pone.0203864

**Published:** 2018-09-25

**Authors:** Clovis Nkoke, Engelbert Bain Luchuo, Ahmadou Musa Jingi, Christelle Makoge, Ba Hamadou, Anastase Dzudie

**Affiliations:** 1 Buea Regional Hospital, Buea, Cameroon; 2 Clinical Research Education, Networking and Consultancy, Douala, Cameroon; 3 Athena Institute for Research on Innovation and Communication in Health and Life Sciences, Vrije Universiteit, Amsterdam, The Netherlands; 4 Faculty of Medicine and Biomedical Sciences, University of Yaounde1, Yaounde, Cameroon; 5 Douala General Hospital, Douala, Cameroon; Hospital das Clinicas da Universidade Federal de Minas Gerais, BRAZIL

## Abstract

**Background:**

Early diagnosis and adequate treatment of Group A streptococcal throat infection is an important initial stage in the primary prevention of acute rheumatic fever and rheumatic heart disease. This preventable condition associated with high mortality rates mandates a thorough understanding by the general public and the health.

**Objective:**

The aim of the study was to assess the level of awareness about different aspects of rheumatic heart disease in patients coming to the outpatient department of the Buea regional Hospital, South West region of Cameroon.

**Methods:**

This was a cross-sectional descriptive study carried in the outpatient department of the Buea Regional Hospital, Cameroon. The study population was adults and children aged 9 years and above. Data collection was done by using a self-administered questionnaire addressed to assess awareness on rheumatic heart disease.

**Results:**

A total of 256 participants were interviewed, of which 70 (27.3%) were males. Their mean age was 34.4 ± 11.9 years (males: 36.2 ± 12.7 years versus females: 33.7 ± 11.6 years, p = 0.129). Most of the participants were in the 20 to 29 year old group (37.9%). More than two thirds (71.1%) of the participants reported having had sore throat at least once. The disease was treated with antibiotics in only 45.4%, with the treatment prescribed by a health care professional in 35.8% of the cases. About 73% of the respondents did not know what causes sore throat, and most (71.1%) were unaware of any complications that could arise from poorly treated sore throat. More than 70% of the participants did not know that sore throat can be associated with heart disease. Rheumatic heart disease was unknown to 82% of the participants and 95% of them did not know what causes RHD. Only 5.1% percent of the participants had an adequate knowledge of RHD. Age ≤ 35 years, post-secondary level of education, and having heard of RHD were significantly associated with an adequate knowledge. After adjusting for age, post-secondary education (aOR: 9, [95% C: 1.2–67.5], p = 0.019), and having heard of RHD (aOR: 18.1, [95% CI: 4.7–70.3], p<0.001) were still associated with a fair knowledge.

**Conclusions:**

Levels of knowledge and awareness on rheumatic heart disease is low. This study provides important insight into the perception and practices related to sore throat that can be used in the design of awareness activities aimed reducing the risk of RHD in Cameroon. The appropriateness of antibiotics prescribed, and the health care provider awareness and knowledge levels regarding RHD in Cameroon has not been reported yet in the literature. This grey area deserves more research.

## Introduction

Rheumatic heart disease (RHD) has virtually disappeared in developed countries but prevails as a major public health issue in children and young adults in low and middle income countries including Cameroon where it causes significant morbidity and mortality [1–4]. It is the leading cause of heart failure in children and young adults in Africa [1]. In Sub-Saharan Africa (SSA), echo based screening studies have consistently shown rates of 1–3% in school children [5–8]. In Cameroon, the prevalence of rheumatic heart disease from hospital based echocardiographic studies has been shown to range from 6–9% [9, 10].

Rheumatic heart disease results from repeated acute rheumatic fever attacks (ARF) subsequent to Group A streptococcal (GAS) throat infections. Early diagnosis and adequate treatment of Group A streptococcal throat infection is an important initial stage in primary prevention of ARF. Poorly treated streptococcal sore throat leads to ARF in a small proportion of patients and about half of patients with acute rheumatic fever will go on to develop rheumatic heart disease. There is no national RHD prevention program in Cameroon. Primary prevention was recognized to be a key component of successful national public health initiatives in several countries where RHD was common [11, 12]. Control and prevention of this deadly disease requires thorough understanding by the general public, health personnel and the patient population. Very little is known of populations’ knowledge about the disease in our country. In order to inform relevant and effective campaign messaging, we sought to understand baseline knowledge about different aspects of RHD in Buea, a semi urban setting in the South West region of Cameroon.

## Materials and methods

### Study design and setting

This was a cross-sectional study conducted in the outpatient department of the Buea Regional hospital, South West region of Cameroon. Buea (a semi-urban setting) is the regional capital of the South West region of Cameroon, in sub-Saharan Africa. The regional hospital is a third level reference hospital with a bed capacity of 200, and a catchment population of 130000 inhabitants. Besides the care of patients, it serves as a teaching hospital of the Buea University. This study was carried out in the first week of May 2017

### Participants and data collection

These were adults and older children of both sexes (>9 years), who were seen at the out-patient department for various reasons (consultation, patient assistance), and who consented to participate in the study. Verbal informed consent was taken from all the patients. The participants were briefly informed about the objective and benefits of participating in the study as they gathered in the waiting room of the outpatient department of the hospital. Each participant was approached individually, both patients and those accompanying patients. Participants were mostly ambulant. The population answering the questionnaires did not necessarily have clinical or subclinical RHD, nor past history of ARF or family history of RHD / valve disease. We excluded people who were critically ill, people who came in with emergency medical conditions.

### Variables

Each participant had to fill in his/her questionnaire independently under direct observation of the investigator or under guidance in the case of children and those who didn't understand the questions and needed help. The survey was conducted in a period of one week to prevent dilution of the questions. The questionnaire compromised of two parts: The first part was about the demographic profile of the participants and the second part was about the awareness regarding the disease, its treatment and complications. The questionnaire was administered by a cardiologist (CN) and two final year medical students with good knowledge of ARF and RHD.

Most of the questions were simple 'yes' 'no' and ‘don’t’ answers.

### Sample size

A consecutive sample of all consenting participants was considered for this study, as this was the first of its kind in our setting, to the best of our knowledge. During the week the survey was conducted, 335 persons came to the outpatient department according to the outpatient register.

### Data analysis

The data collected was entered in Microsoft excel. We analyzed the data using the software Epi Info version 7. We derived a three item score to assess the level of awareness of RHD from this statement: Sore throat can be caused by a bacterium (Yes = 1 point, No or don’t know = 0 point). Untreated sore throat can be associated with heart disease (Yes = 1 point. No or don’t know = 0 point). Proper treatment of sore throat can prevent Heart Disease (Yes = 1 point. No or don’t know = 0 point). A total score of zero = very poor knowledge, 1 = poor knowledge, 2 = Fair knowledge, and 3 = Adequate knowledge. We assessed for factors that can be associated with adequate knowledge such as: age, sex, post-secondary level of education, history of sore throat, and ever heard of RHD. We present these as Odds Ratios (OR). We present continuous variables as means ± standard deviation, and discrete variables as frequencies and percentages with their 95% Confidence intervals. A p value <0.05 was considered statistically significant for the observed associations.

### Ethics statement

This study was approved by the administrative authorities of the Buea Regional Hospital acting as the local ethics committee. We carried out this study in accordance with the declarations of Helsinki. We report this work according to standards for reporting epidemiological studies (STROBE) guidelines.

Verbal informed consent was approved by the hospital ethics committee. Guardian consent was collected for all participants under age 18. The study was explained in Pidgin English which is understood by all to ensure comprehension. Participants were informed they were free not to participate in the study, or to discontinue answering the questions at any point during then study, without any impact on the health services they received in the health facility. Confidentiality and privacy of information was assured.

## Results

### Socio-demographic characteristics

A total of 256 participants were interviewed, of which 70 (27.3%) were males. Their mean age was 34.4 ± 11.9 years (males: 36.2 ± 12.7 years versus females: 33.7 ± 11.6 years, p = 0.129). Most of the participants were in the 20 to 29 year old group (37.9%). The majority of the participants had a post secondary level of education (44.5%).

### Awareness about different aspects of rheumatic heart disease

The characteristics of the study population are shown in Table 1. More than two third (71.1%) of the participants reported having had sore throat at least once. The disease was treated with antibiotics in only 45.4%, with the treatment prescribed by a health care professional in 35.8% of the cases. About 73% of the respondents did not know what causes sore throat, and most (71.1%) were unaware of any complications that could arise from poorly treated sore throat. More than 70% of the participants did not know that sore throat can be associated with heart disease. Rheumatic heart disease was unknown to 82% of the participants and 95% of them did not know what causes RHD. Only 5.1% of the participants had an adequate level of awareness of rheumatic heart disease. Factors associated with an adequate knowledge of RHD are shown in Table 2. Age ≤ 35 years, post-secondary level of education, and having heard of RHD were significantly associated with an adequate knowledge. After adjusting for age, post-secondary education (aOR: 9, [95% C: 1.2–67.5], p = 0.019), and having heard of RHD (aOR: 18.1, [95% CI: 4.7–70.3], p<0.001) were still associated with a fair knowledge (Table 2).

**Table 1:** Socio-demographic characteristics and awareness of rheumatic heart disease.

Variable	Frequency (N)	Percentage, % (95% C I)
**Sex**		
Male	70	27.3 (22–33.2)
Female	186	72.7 (66.8–78)
**Level of Education**		
Primary	68	26.6 (21.3–32.4)
Secondary	74	28.9 (23.4–34.9)
Post-Secondary	114	44.5 (38.3–50.9)
**History of Sore throat**		
Yes	182	71.1 (65.1–76.6)
No	74	28.9 (23.4–34.9)
**Treatment used to treat sore throat (N = 172)**		
Anti-biotics	78	45.4 (37.8–53.1)
Gargling of Salt	43	25 (18.7–32.2)
Use of herbs	26	15.1 (10.1–21.4)
Others	25	14.5 (9.6–20.7)
**Who prescribed treatment? (N = 162)**		
Self-medication	47	29 (22.2–36.7)
Friend/Relative	57	35.2 (27.9–43.1)
Health Care Professional	58	35.8 (28.4–43.7)
**What causes sore throat?**		
Bacteria*	39	15.2 (11.1–20.2)
I don’t know	188	73.4 (67.6–78.7)
Others	29	11.3 (7.7–15.9)
**Do you know any complication of untreated sore throat?**		
Yes	74	28.9 (23.4–34.9)
No	182	71.1 (65.1–76.1)
**Can sore throat be associated with heart disease?**		
Yes*	48	18.8 (14.2–24.1)
No	28	10.9 (7.4–15.4)
I don’t know	180	70.3 (64.3–75.8)
**Can treating sore throat prevent Heart disease?**		
Yes*	93	36.3 (30.4–42.6)
No	71	27.7 (22.3–33.7)
I don’t know	92	35.9 (30.1–42.2)
**Have you heard of Rheumatic Heart Disease (RHD)?**		
Yes	43	16.8 (12.4–22)
No	213	83(78.1–87.2)
**Level of awareness of RHD**		
Very poor	142	55.5 (49.2–61.7)
Poor	61	23.8 (18.7–29.5)
Fair	40	15.6 (11.4–20.7)
Adequate	13	5.1 (2.7–8.5)

**Table 2:** Factors associated with adequate knowledge of RHD.

Variable	Odds Ratio (95% CI)	p value
Sex		
Male	0.21 (0.03–1.64)	0.103
Female	1	
Age		
≤ 35 years	8.8 (1.13–69)	0.013
>35 years	1	
Post-secondary Level of education		
Yes	16.6 (2.1–129.6)	0.0004
No	1	
History of sore throat		
Yes	2.3 (0.5–10.7)	0.269
No	1	
Heard of Rheumatic Heart Disease		
Yes	21.2 (5.6–81.1)	<0.001
No	1	

## Discussion

To the best of our knowledge this is the first survey of RHD awareness in Cameroon. In this survey, our results show that the level of awareness of RHD is low. Factors associated with adequate knowledge of RHD were age ≤ 35 years, post-secondary level of education, and having heard of RHD.

Rheumatic heart disease is the leading cause of acquired heart disease among the young worldwide [3]. It is a major cause of heart failure in children and young adults in Africa [13]. It results in premature death, with many requiring surgery which is usually not affordable by most families. Rheumatic heart disease results from repeated ARF attacks following exposure to Group A streptococcal (GAS) infections. It is a disease that is 100% preventable.

The current strategies for the prevention for RHD includes: improvement of socioeconomic conditions with better sanitation and housing (i.e. primordial prevention); primary prevention through antibiotic treatment of GAS pharyngitis; and secondary prevention of ARF recurrence by penicillin prophylaxis against repeated or chronic ARF attacks [14]. Early diagnosis and adequate treatment of Group A streptococcal throat infection is an important initial stage in primary prevention. This will require adequate knowledge of RHD by the general public and health care professional including doctors and nurses. In this study, the level of awareness of RHD was very low with only 5% of the participants having an adequate knowledge about the different aspects of the disease. Even in a population of patients affected by RHD, knowledge about RHD was low [15]. In their study, *Saeed et al* reported that only 5% of patients with RHD were aware that sore throat was the cause of the disease [15]. Even doctors’ awareness about prevention of ARF and RHD in Sudan was average. This was later on raised by intervention through lectures to good levels [16]. Only 18.8% of the participants in our study were aware that sore throat is a precipitating factor for RHD. This was however higher than that reported in patients with RHD where only 5% were aware of this association [15]. In our study, 71.1% of the participants reported having had a sore throat at least once. This was much higher than that reported in school children in Zambia [17].

Streptococcal sore throat should be treated with antibiotics within 9 days from onset to eliminate the risk of RHD [18]. Regarding treatment practices, the management of sore throat in our study was sub-optimal with less than half of those who had a previous sore throat receiving antibiotics, indicating a high risk of developing ARF and subsequent RHD. This proportion was very close to that reported in Zambia [17]. Close to half of the participants used salt gargling and traditional herbs for the treatment of sore throat. In a study in Tanzania, factors that limited the diagnosis and treatment of GAS pharyngitis was the fact that patients did not present for treatment of sore throat; and that there was little patient and community knowledge regarding the importance of treating a sore throat [19]. In South Africa, a concerningly high number of patients with RHD had poor knowledge between sore throat and RHD and preferred local remedies or simple pain medications [20].

The burden of RHD can be reduced through primary, secondary and tertiary prevention intervention programs. The current focus of global efforts at prevention of RHD is on secondary prevention in the form of regular administration of penicillin to prevent recurrent episodes of ARF. However in low and middle income countries, valvular damage due to previous unrecognized episodes of rheumatic fever has already occurred by the time secondary prophylaxis is instituted. General public awareness activities are vital for a successful RHD control program [21]. Awareness activities was able to improve awareness of RHD in Nepal by 40% (from 8% to 48%) [22]. The Nepal Heart Foundation used a wide variety of activities to improve awareness of RHD such as putting large hoarding boards throughout the cities; mobilizing the media; including RHD materials in school curriculums; showing street dramas; distributing pamphlets, posters, and calendars [22]. Such awareness activities should be advocated in Cameroon through a RHD control program as the level of awareness of ARF/RHD was comparable to that in Nepal [22]. Improvement of awareness of RHD will encourage people to seek medical attention for a streptococcal throat infection and treat it with a suitable antibiotic, thus reducing the risk of developing ARF/RHD.

Community programs have resulted in the virtual elimination of rheumatic fever in Cuba. This was achieved using comprehensive, integrated programs targeting primary and secondary prevention [23]. The program that stretched over 10 years was able to demonstrate a reduction in rheumatic fever from 18.6 per 100,000 to 2.5 per 100,000. These were both comprehensive community interventions, consisting of awareness campaigns, establishment of registries and medical training with particular emphasis on primary and secondary prevention. Hence a strategy that includes primary prevention can be markedly effective in preventing rheumatic fever and RHD. In Africa, the ASAP (Advocacy, Surveillance, Awareness, and Prevention) program, under the auspices of the Pan-African Society of Cardiology has galvanized efforts in Africa to combat this disease [24].

Primary prevention through antibiotic treatment of GAS prevents ARF/RHD and penicillin prevents RHD progression when initiated in a timely manner. As a result, early detection has been highlighted to be of particular interest [2]. Reports suggest that focused cardiac ultrasound by non experts using pocket devices seems feasible and yields acceptable sensitivity and specificity for RHD detection, opening new perspectives for mass screening for RHD in resource limited settings where there is scarcity of skilled personnel and standard echocardiography [25]. In our study, less than half of the participants who reported an episode of sore throat were treated with antibiotics, indicating a risk of having ARF/RHD. With the low level of awareness, awareness activities coupled with such mass screening campaigns with pocked devices can lead to early detection of RHD to better prevent adverse outcomes.

## Conclusion

The results of this study show that the population is lacking knowledge about all aspects of RHD and its treatment. This may have a profound effect on the incidence of rheumatic disease in the setting. Our results can be used in the design of awareness activities aimed reducing the risk of RHD in Cameroon. The appropriateness of antibiotics prescribed, and the health care provider awareness and knowledge levels regarding RHD in Cameroon has not been reported yet in the literature. This grey area deserves more research.

## Supporting information

S1 RHD awareness questionnaire (DOCX)

S1 RHD database (XLSX)
