# Supplementary material for: Rheumatic heart disease awareness in the South West region of Cameroon: A hospital based survey in a Sub-Saharan African setting
Source: PLoS One. 2018 Sep 25;13(9):e0203864. doi: 10.1371/journal.pone.0203864 (PMC6155442; doi:10.1371/journal.pone.0203864)
Supplement: S1 RHD awareness questionnaire — (DOCX) [file pone.0203864.s001.docx]

**Awareness of rheumatic heart disease in a semi-urban setting in Cameroon**

1. **Socio-demographic data**
2. Age_________________________________________________________
3. Sex__________________________________________________________
4. Occupation____________________________________________________
5. Level of education
   1. Primary school
   2. Secondary school
   3. Post secondary school
6. **Awareness regarding rheumatic heart disease**
7. Have you ever had a sore throat?_______________________ (**yes=1, no=2**)
8. Has any of your children ever had a sore throat_____________(**yes=1, no=2**)
9. If yes in question 1 and/or 2: what did you do to treat the sore throat? __________________________________________________________________

(1= Antibiotics, 2= salt, 3=traditional herbs, 4=0thers)

1. Who prescribed medication when you or a child had a sore throat?____________

(My self =1, Friend=2, Medical doctor/health care professional=3)

1. Do you have any family member or know someone who frequently has sore throat (more than 3 times are year)? Yes = 1, no =2
2. In your opinion what causes sore throat? **I don’t know=1, Bacteria=2, Others=3**
3. Are you aware of any complications that could occur in case sore throat is poorly treated? __________(**yes=1, no=2**)
4. Can sore throat be associated with heart disease?__________(**yes=1, no=2 , no idea=3**)
5. Adequate treatment of acute sore throat is an important tool in prevention of heart Disease:_____________________ (**true or false**)
6. Have you ever heard about rheumatic heart disease_____________(**yes=1, no=2**).
7. What causes rheumatic heart disease? ___________________________________
